# Micromorphology and Chemical Studies on *Anacardium occidentale* L. Stem Bark as an Herbal Medicine

**DOI:** 10.3390/plants12010007

**Published:** 2022-12-20

**Authors:** Sofia Encarnação, Rita Serrano, Cristina Almeida, Olga Silva

**Affiliations:** Research Institute for Medicines (iMed.ULisboa), Faculty of Pharmacy, Universidade de Lisboa, 1649-003 Lisbon, Portugal

**Keywords:** *Anacardium occidentale*, assay, chromatographic fingerprint, histochemistry, identification, medicinal plants, microscopic analysis, quality control, stem bark

## Abstract

The red and white types of *Anacardium occidentale* L. stem barks (AoB) are used in the Community of Portuguese Language Countries, including Portugal, to make traditional herbal preparations for the treatment of diabetes. This work aims to obtain the macroscopic, micromorphological, chemical, and purity data necessary to use both types of AoBs as medicinal plants safely. Macroscopically, a brown inner (red AoB) or a dark-brown inner surface (white AoB) was observed. Light and scanning electron microscopy showed that sclereid cells with thin cell walls were significantly larger (*p* < 0.001) in red AoB than in white AoB, but calcium oxalate druses and starch grain areas were significantly larger (*p* < 0.001) in white AoB than in red AoB. The chromatographic profiles (thin-layer chromatography and high-performance liquid chromatography) of both types of AoBs were characterized by the presence of gallic and protocatechuic acids and other phenolic derivatives. The condensed tannins were the major secondary metabolites class (143.69 ± 4.67 mg and 73.79 ± 4.46 mg catechin equivalents/g AoB in red and white types, respectively). The loss on drying and the total ash were, respectively, 7.07 ± 0.16% and 2.31 ± 0.18% in red AoB and 6.58 ± 0.16% and 1.94 ± 0.14% in white AoB. The obtained data are helpful in ensuring the quality of AoB as raw material for the pharmaceutical market.

## 1. Introduction

The *Anacardium occidentale* L. is one of the 20 species of the *Anacardium* genera and belongs to the *Anacardiaceae* Lindl., a family that includes about 77 genera and 700 species [1]. According to The World Flora Online, *Anacardium occidentale* is the name currently accepted to refer to this species, and *Acajuba occidentalis* (L.) Gaertn., *Anacardium microcarpum* Ducke, *Cassuvium pomiferum* Lam., *Cassuvium reniforme* Blanco, *Cassuvium solitarium* Stokes are its botanical synonyms [2].

Commonly known as cashew (English), cajú (Portuguese), or caju (Crioulo), *A. occidentale* is a native plant to South America [2,3,4]. The cashew was introduced to the East by the Portuguese in the 16th century [4]. Nevertheless, the introduction of cashew in West Africa is relatively recent [5], and the tree has been mainly naturalized in its coastal districts [6]. Currently, cashew is widely cultivated in vast orchards in Latin American, African, and Asian countries such as Brazil, Cuba, Guinea-Bissau, Mozambique, and India [3].

The cashew was initially used in Africa as a fire protection barrier around forest demarcations [7]. Nowadays, the African continent is among the main cashew production areas, and its cultivation is a strategic point for the economic development of the continent [8].

The cashew is described in different Floras in which the plant was introduced (e.g., Brazilian Flora, Flora of West Tropical Africa, Flora of Central Africa, Flora Zambeziaca, Flora Malesiana, and Flora of China). Still, no description has been performed in Flora of Europe [2,6].

*A. occidentale* is an evergreen tree that grows up to 15 m and has a short, thick, and tortuous trunk [9]. In “Flora of West Tropical Africa,” *A. occidentale* was described as a plant with angular and glabrous branchlets. The leaf is simple, obovate, or oblong-obovate, cuneate at the base, rounded or truncate at the apex (8–15 cm), long (5–10 cm), broad, glabrous, and closely reticulate, with 10–15 pairs of lateral nerves. The petiole is long (up to 2 cm) and flat above; the flower is polygamous in lax terminal panicles; the bracts are large, enfolding the flowers, and are ovate, acuminate, and softly puberulous; the flower presents five linear petals and ten stamens (only some have anthers). The fruit is a kidney-shaped nut that grows at the end of a false fruit, commonly called an “apple” [6]. This false fruit develops from the receptacle of the cashew flower and is fleshy, juicy, yellowish or reddish, and pear-shaped [7,9]. The red and white types of *A. occidentale* are recognized according to the false fruit’s color [10].

The cashew tree supplies wood, which is helpful in local economies, but most cultivation is directed toward nut crop production [11,12]. In addition, different parts of this plant species are used in traditional herbal preparations (THPs). The bark is used for a wide range of therapeutic purposes, namely to treat external and internal wounds, stomachache, cough, toothaches, diarrhea, hypertension, diabetes, hemorrhoids, and sexual dysfunction [1,13,14,15] during all seasons. However, the false fruits only occur during the fructification time, so it is difficult to distinguish the various types of plants outside that period [10].

Different chemical classes of secondary metabolites, including flavonoids, coumarins, tannins, other phenol acid derivatives, and triterpenoids were reported in *Anacardium occidentale* stem bark (AoB) [16,17,18,19,20]. Catechin, epicatechin, epigallocatechin, gallic acid, stigmast-4-en-3-one, and tanacetene are examples of compounds already identified in this medicinal plant, whose nut and false fruit are edible [21,22,23].

A Portuguese traditional herbal preparation (THP) based on red and white types of AoB aqueous extracts is clinically used by oral administration to control type 2 diabetes [24]. Pharmacological assessments for safety and efficacy have been performed with this THP [17]. Furthermore, a preliminary phytochemical analysis of red and white AoBs was carried out by using thin-layer chromatography (TLC) [25], and the total phenolic content was determined by a colorimetric assay [17]. Additionally, gallic acid, protocatechuic acid, and ellagic acid were identified on the red AoBTHP [26].

It should be noted that 70–95 percent of the population uses traditional medicine for primary health care in Africa, America, and Asia [27]. In Europe, over 100 million people also frequently use traditional medicine to complement allopathic medicine [28].

The World Health Organization established a strategy to promote the safe and effective use of traditional medicine, which involved regulating products, practices, and practitioners [29]. Since there are no reports of systematic pharmacognostical studies on the red and white AoBs, the present work was carried out to study the detailed macro- and microscopic characteristics of the dried whole, fragmented, and powdered AoBs. This study also aims to provide an AoB qualitative and quantitative chemical analysis using histochemical, chromatographic, and spectrophotometric assays and purity tests.

## 2. Results

### 2.1. Macroscopy

**Figure 1:**
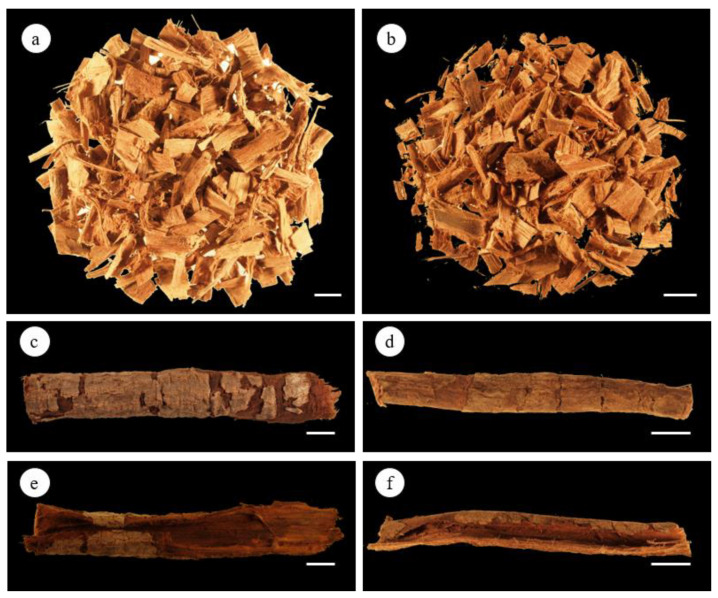
*A. occidentale* stem bark macroscopic characteristics. (**a**) General aspect of white AoB; (**b**) general aspect of red AoB; (**c**) inner surface of white AoB; (**d**) inner surface of red AoB; (**e**) outer surface of white AoB; (**f**) outer surface of red AoB. Scale bar = 1 cm.

The general aspects of the white and red AoB samples are presented in Figure 1a,b. The AoBs showed a single quill shape or was curved and slightly concave on the inner side (Figure 1c–f). The selected samples had variable lengths, widths, and thicknesses, as shown in Table 1.

**Figure 2:**
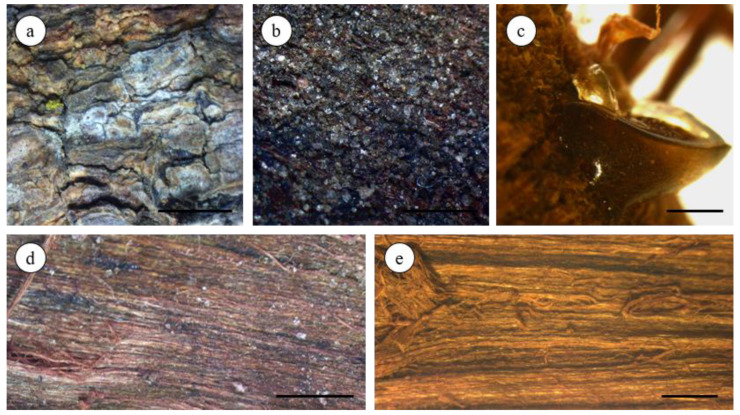
Stereomicroscopy photographs of *A. occidentale* stem bark. Outer surface with (**a**) greyish lichens and yellow moss; (**b**) calcium oxalate crystals; (**c**) topaz-colored gum resin; (**d**) dark brown inner surface of white AoB; (**e**) reddish-brown inner surface of red AoB. scale bar: (**a**,**d**,**e**) = 2 mm; (**b**) = 500 µm; (**c**) = 1 mm.

The outer surface was greyish brown, bearing greyish lichens, yellow moss, and calcium oxalate crystals (Figure 2a,b). It produced an exudate, which was a shiny, topaz-colored gum resin (Figure 2c). The inner surface was dark brown in white AoB and reddish-brown in red AoB (Figure 2d,e). The bark was finely striated in the longitudinal direction, and its fracture was fibrous. The AoBs had a slightly aromatic characteristic odor and an astringent taste.

### 2.2. Microscopy

**Figure 3:**
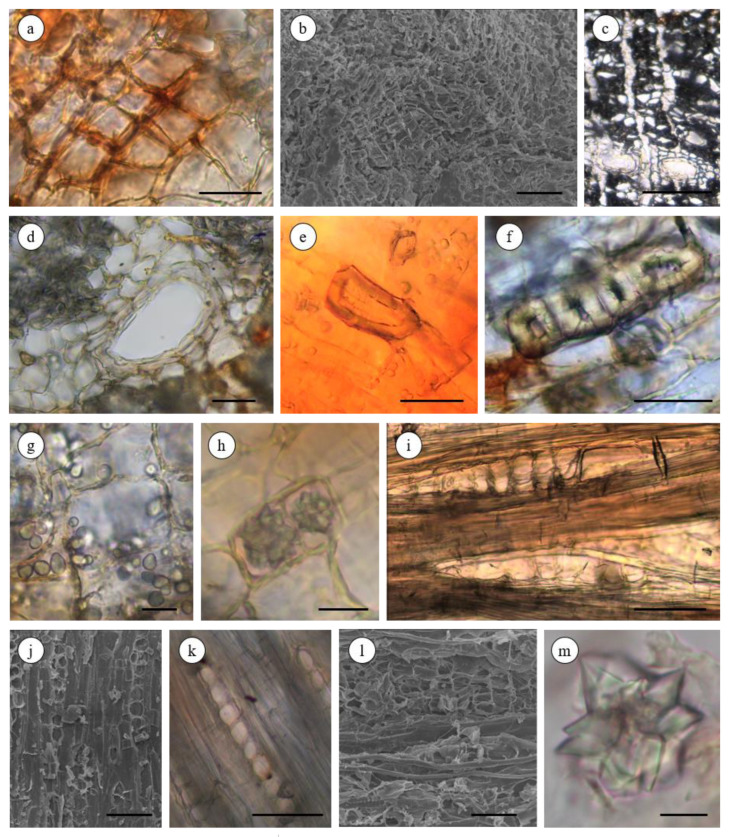
Light and scanning electron microscopy photographs of *A. occidentale* stem bark (**a**,**c**–**h**) and scanning electron microscopy (**b**) photographs of transverse sections of AoB; (**a**,**b**) flattened and reddish-brown phellem cells, with slightly thickened walls; (**c**) phelloderm cell tissue and cortical parenchyma with secretory channels; (**d**) secretory channel; (**e**) thin-walled sclereids. (**f**) Group of thick-walled sclereids; (**g**) starch granules; (**h**) idioblast containing calcium oxalate crystal clusters. Light microscopy (**i**,**k**,**m**) and scanning electron microscopy (**j**,**l**) photographs of longitudinal sections of AoB; (**i**–**k**) uni-, bi-, tri- and tetra-seriate medullary rays; (**k**,**l**) sieve tubes, phloem parenchyma, and phloem fibers. (**m**) Light microscopy photograph of an isolated druse of calcium oxalate. Scale bar: (**a**,**c**–**f**) = 50 µm; (**g**,**h**) = 20 µm; (**b**,**i**–**l**) = 100 µm; (**m**) = 10 µm.

Microscopy examination of a typical transverse section of the stem bark of *A. occidentale* by light microscopy (LM) and scanning electron microscopy (Figure 3a–h) showed 4–6 layers of flattened and reddish-brown phellem cells with slightly thickened walls (Figure 3a,b). The area values of the different anatomical structures of AoBs examined by LM are presented in Table 2.

The phelloderm cell tissue and cortical parenchyma made up of several layers of flat, polygonal, and thin-walled cells were also observed (Figure 3c). This parenchyma exhibited irregularly arranged secretory channels (Figure 3c,d) and two forms of sclereids: thin-walled sclereids (Figure 3e) and small groups of thick-walled sclereids (Figure 3f). In the cortex were also observed small, simple, and spherical starch granules (Figure 3g) and clusters of calcium oxalate crystals. Idioblasts containing calcium oxalate crystal clusters (Figure 3h) were found scattered in the layers of the cortex. The examination of the parenchyma showed that the white AoB had a much thicker cortical parenchyma than the red AoB.

LM and scanning electron microscopy of bark longitudinal sections (Figure 3i–l; Table 2) revealed typical uni-, bi-, tri-, and tetra-seriate medullary rays (Figure 3i–k), sieve tubes, phloem parenchyma, and phloem fibers (Figure 3k,l). The phloem was composed of dense tissues and fibers of sclerenchyma with thin walls that alternate with bands of phloem parenchyma interspersed with sieve cells. The phloem fibers were many and large, and usually occurred in groups. Narrow medullary rays crossed the phloem parenchyma and were one to three cells wide. The area of sclereid cells with thin cell walls was statistically significantly higher (*p* < 0.001) in red AoB (1370.51 ± 100.89 μm^2^) than in white AoB (955.27 ± 117.21 μm^2^). The areas of druses of calcium oxalate and starch grains were statistically significantly higher (*p* < 0.001) in white AoB (575.17 ± 102.03 μm^2^ and 18.25 ± 2.73 μm^2^, respectively) than in red AoB (279.32 ± 34.04 μm^2^ and 9.43 ± 1.54 μm^2^, respectively). Nevertheless, no statistically significant differences (*p* > 0.05) were observed in the other assessed morphological and anatomical characteristics of both types of AoBs.

### 2.3. Powder

The powder of AoB exhibited a reddish-brown color with a characteristic odor and a bitter taste. Microscopically, it was characterized by the features mentioned earlier and illustrated structures. The most common structures observed were fragments of phloem fibers (Figure 3k), phellem cells (Figure 3a), medullary ray cells (Figure 3i–k), parenchymatous cells containing small and spherical starch granules (Figure 3g), isolated druses of calcium oxalate (Figure 3m), and rarely, calcium oxalate prisms.

### 2.4. Histochemical Tests

Using LM and chromatic staining reactions, phenols, tannins, triterpenoids, and starch were identified on both types of AoBs (Table 3).

### 2.5. Chemical Studies

#### 2.5.1. Drug-Extract Ratio of Aqueous Extracts

The drug-extract ratio of THP was 1:7.92 for white AoB and 1:7.62 for red AoB.

#### 2.5.2. Thin-Layer Chromatography

**Figure 4:**
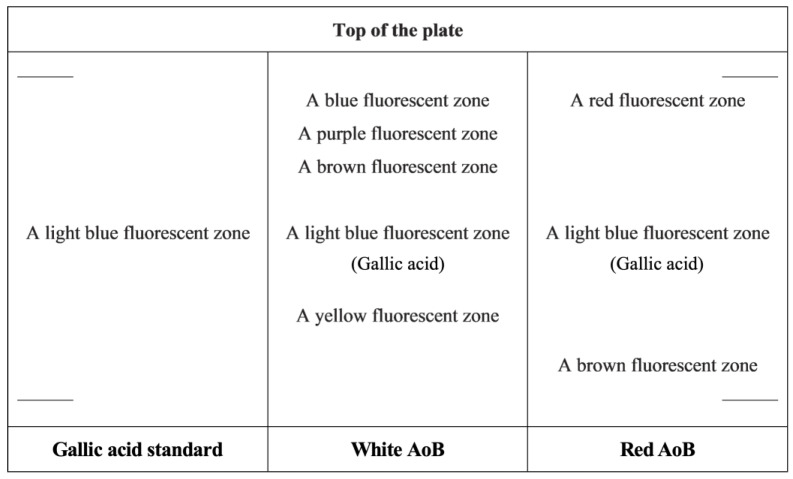
White and red AoB TLC fingerprint schemes.

Following the analytical monographic sequence preconized on the actual edition of the European Pharmacopoeia, after macro- and microscopic botanical identification, the chemical profiles of red and white AoBs were established by TLC. Results showed for both types of AoBs a characteristic light blue, fluorescent zone at a retention factor (R_f_) of about 0.58, corresponding to gallic acid, together with a group of distinctive fluorescent zones corresponding to other phenolic acid derivatives (Figure 4).

#### 2.5.3. LC-UV Analysis

**Figure 5:**
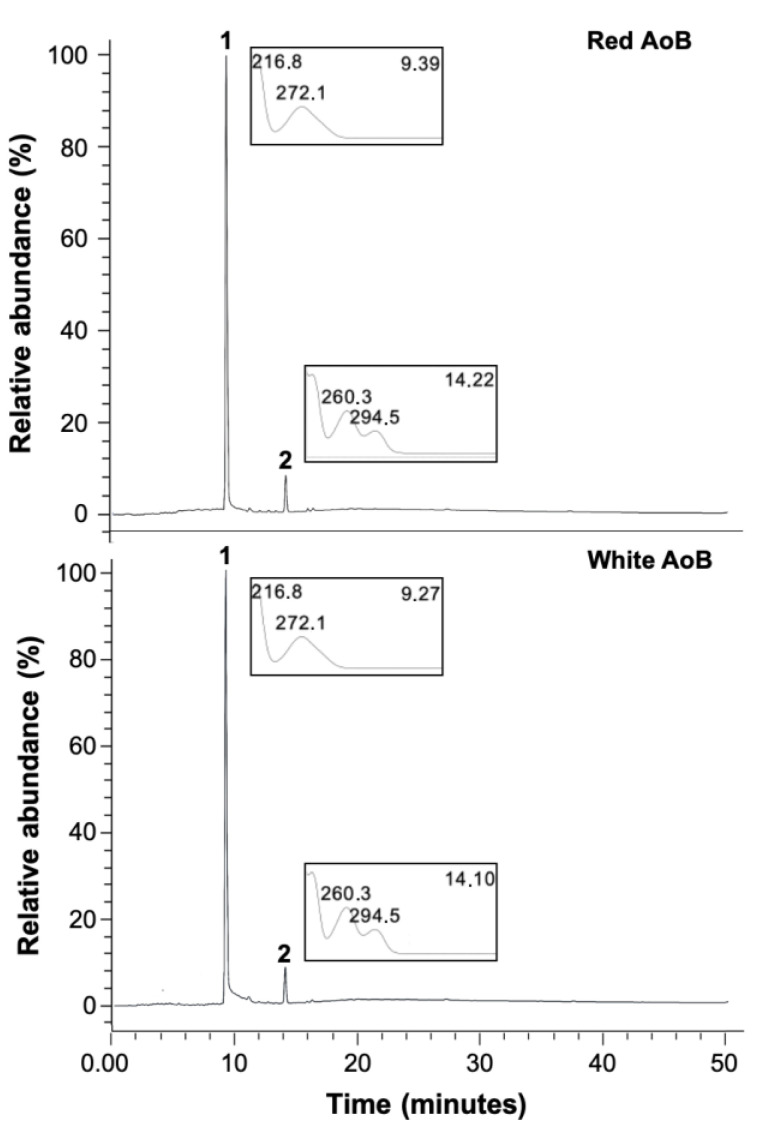
Representative LC-UV/DAD chromatographic profiles for the red and white AoBs. 1: gallic acid; 2: protocatechuic acid.

Chromatograms of red and white AoB extracts obtained by high-performance liquid chromatography (LC) coupled to an ultraviolet photodiode array detector (UV/DAD) are presented in Figure 5. By co-chromatography, gallic acid and protocatechuic acid were confirmed as markers and major compounds of both types of AoBs. Concerning the red AoB, obtained results were similar to those previously obtained by us with another sample of the same medicinal plant, except for ellagic acid, which was not detectable now [26].

#### 2.5.4. Quantification of Secondary Metabolites by Spectrophotometry

The contents of the total phenols, hydrolyzable tannins, condensed tannins, and total triterpenoids in red and white AoBs are presented in Table 4.

Both types of AoBs have condensed tannins and triterpenoids as their main secondary classes of metabolites and hydrolyzable tannins as their less abundant secondary metabolites.

The total phenol content was similar in red and white AoBs. The condensed tannin content was statistically significantly higher (*p* < 0.0001) in red AoB than in white AoB. However, the contents of hydrolyzable tannins and total triterpenoids were statistically significantly higher (*p* < 0.0001) in white AoB than in red AoB.

### 2.6. Tests

#### 2.6.1. Loss on Drying

The loss on drying was 7.07 ± 0.16% in red AoB and 6.58 ± 0.16% in white AoB.

#### 2.6.2. Total Ash

The total ash of the red and white AoBs were 2.31 ± 0.18% and 1.94 ± 0.14%, respectively.

## 3. Discussion

The vital role played by medicinal plants in healthcare and the rising interest in traditional medicine have contributed to the growing concern about the authenticity of plant material to ensure its quality, safety, and efficacy [30,31].

The occurrence of adverse events is frequently attributed to quality problems, so the establishment of raw plant material quality criteria is crucial and mandatory [32].

In official media, plant identification is primarily made by botanical characterization, and phytochemical characterization is complementary data [33]. Botanical microscopy is assumed as an easy, objective, and low-cost quality assessment tool for the physical examination of botanicals and their authentication [33].

Nevertheless, there is no monograph for *A. occidentale* with the definition of macro- and microscopic botanical characteristics, as well as chromatographic characteristics obtained from TLC and LC-UV/DAD to identify and authenticate its stem bark. Until our work, there was also no information about the botanical features of the identification of red and white types of AoBs. Only one preliminary macro- and microscopic botanical characterization was performed on AoB by Leopoldino Antão, and no mention of its type or origin was made [9]. Furthermore, no study has provided data on AoB combining histochemical tests and TLC.

Macroscopic and microscopic characteristics of AoB allowed the definition of bark structural features for its botanical characterization and the distinguishing of red and white types of *A. occidentale*.

The presence of tannin secretory channels is a registered microscopic anatomical feature that is characteristic of the *Anacardiaceae* family. These compounds are usually produced in the parenchyma [34]. The observation of a little-developed phelloderm and the abundant presence of oxalate calcium crystals were already reported in other species belonging to the *Anacardiaceae* [34]. In this work, it was observed that the areas of sclereid cells with thin cell walls, druses of calcium oxalate, and starch grains might be used as differentiating characteristic features of the red and white AoBs. It was determined that red AoB presented sclereid cells with thin cell walls and starch grains with significantly larger areas than the white AoB. However, the white AoB had calcium oxalate druses significantly larger than the red AoB. The other features had no considerable variances between the two types of AoBs, so that they may serve as diagnostic features for plant identification.

Microscopic examination revealed the absence of notable differences in the features of the red and white AoB powders. Thus, these cannot be used to distinguish between the two types of AoBs.

The detection of phenolic acids and hydrolyzable and condensed tannins in situ by histochemistry was in accordance with the TLC results and the previous phytochemical studies conducted by our team [17].

In comparison with the results previously obtained by our team (THP drug-extract ratio of 14:1 for red AoB and 12:1 for white AoB), the drug-extract ratio of red and white AoBTHP (1:7.62 for red AoB and 1:7.92 for white AoB) was lower, despite the plant material being collected from the same region. However, the collection was carried out in different seasons, a factor of variability that can justify these results.

Our results of total phenolic contents of red and white AoBTHPs (31.39 ± 0.50 mg GAE/g AoB and 31.36 ± 0.54 mg GAE/g AoB, respectively) were distinct from the results obtained by other authors. In fact, Chaves et al. (2010) observed that the total phenolic content of an AoB ethanolic extract was 345.16 ± 16.24 mg GAE/g AoB (plant material origin: Piauií, Brazil; extraction process: maceration) [20]. Madjitoloum et al. (2018) also referred to the total phenolic content of an AoB methanolic extract with the value of 608.52 mg GAE/100 g AoB (plant material origin: Pala Region of Mayo-Kebbi, Chad; extraction process: maceration with mechanical agitation) [35]. These differences can be justified by the different origins of the plant material and distinct methodologies adopted in the studies.

The loss on drying and the total ash of red and white AoB samples followed the requirements established for other bark species, such as *Rhamnus frangula* L. or *Rhamnus purshianus* D.C., in the European Pharmacopoeia 11.0 (maximum 10.0% and 6.0 or 7.0%, respectively) [36].

Regarding the safety and efficacy of the characterized samples and AoBTHPs used in the present study, some data have already been obtained.

These red and white AoBTHPs showed no signs of treatment-related toxicity in CD-1 mice at doses up to 402 mg/kg in a two-week repeated dose toxicity study [17]. A lack of genotoxic potential of both AoBTHPs was observed in a micronucleus test and a comet assay using the CD-1 mouse model [17]. In a 92-day study using a db/db mouse model, red AoBTHPs (40.2, 71.5, and 127.0 mg/kg/day) showed blood-glucose-lowering activity. At 127.0 mg/kg/day doses, the red AoBTHP showed a higher glucose-lowering effect than glibenclamide [26].

## 4. Materials and Methods

### 4.1. Chemicals and Reagents

Acetic anhydride, bismuth nitrate, and sodium nitrite were obtained from Panreac^®^ (Barcelona, Spain). Diphenylboric acid-β-ethylamino ester, fast blue B salt, gallic acid, iodine, oleanolic acid, polyethylene glycol-4000, protocatechuic acid, and vanillin were obtained from Sigma-Aldrich^®^ (Steinheim, Germany). Ethanol was obtained from Aga^®^ (Prior Velho, Portugal). Glacial acetic acid and sulfuric acid were obtained from Chem-Lab^®^ (Zedelgem, Belgium). Acetone, Folin–Ciocalteu reagent, hydrochloric acid 37% extra pure, iron trichloride, perchloric acid, potassium iodate GR, sodium carbonate, sodium hydroxide, and TLC cellulose plates (catalog number 105552) were obtained from Merck^®^ (Darmstadt, Germany). Butanol-1 and methanol were obtained from Fisher Chemicals^®^ (Leicestershire, UK). Ferrous sulfate was obtained from May and Baker LTD^®^ (Dagenham, UK). The (+)-Catechin (hydrate) was obtained from Cayman Chemical Company^®^ (Ann Arbor, MI, USA). Aluminum chloride was obtained from Fluka^®^ (Buchs, Switzerland). Acetonitrile was obtained from Honeywell Riedel-de Haën™ (Seelze, Germany). Formic acid 99–100% was obtained from VWR Chemicals (Fontenay-sous-Bois, France). All used reagents were of analytical grade.

### 4.2. Plant Material

Red and white types of AoBs were collected during the fructification period and dried under shade in Guinea-Bissau. The plant was identified by the collector Luís Catarino at the LISC-Herbarium, Tropical Botanical Garden of Instituto de Investigação Científica Tropical (voucher numbers: white AoB collected at Dulombi, 11.858° N; 14.503° W: LC1924 CJ; and red AoB collected at Paiai, 11.836° N; 14.421° W: LC 1922 LC). The plant material was dried at room temperature, in the absence of direct light, and stored in the Pharmacognosy Laboratory of the Faculty of Pharmacy, “Universidade de Lisboa.” Plant material was homogenized according to the standards of the European Pharmacopoeia 11.0 [37]. Observations were performed on 30 adult red and white bark samples.

According to the European Pharmacopoeia 11.0, the powdered drug used in the botanical identification protocol was obtained by pulverization in a porcelain mortar using a pestle until the coarse powder fineness degree was reached [38].

### 4.3. Macroscopic Analysis

The macroscopic study of the two kinds of AoBs was performed according to the standard methods described in the European Pharmacopoeia 11.0 [39]. The material plant samples were directly examined by the naked eye and by using an Olympus SZ61 stereomicroscope (Switzerland) coupled with a Leica MC170 HD digital camera controlled by the Leica Application Suite (LAS) Version 4.8.0 software (Leica Microsystems Ltd., Heerbrugg, Switzerland). Images were also acquired using this software.

### 4.4. Light Microscopy

The material plant samples were submerged in glycerin 30% to soften the surfaces and allow the sections to be sliced. For anatomical analyses, transverse and tangential longitudinal sections were manually prepared with approximately 1 mm of the underlying thickness, mounted in 60% aqueous chloral hydrate solution, and examined using an Olympus CX31 microscope coupled with a Leica MC170 HD digital camera, controlled by the Leica Application Suite Version 4.8.0 software (Leica Microsystems Ltd., Heerbrugg, Switzerland). Images were also acquired using this software.

### 4.5. Scanning Electron Microscopy

A representative sample of each plant material type (red and white cashew dried bark) was sectioned and directly mounted on stubs using double-sided adhesive tape. Samples were then sputtered with a thin layer of gold in a Polaron E 5350 and observed using a JEOL JSM-T220 scanning electron microscope at 15 kV, with a digital image acquisition integrated system (Peabody, MA, USA).

### 4.6. Histochemical Tests

The chromatic staining reactions were performed in bark transverse sections before examination under LM. The histochemical tests were Fast blue reagent for phenolic compounds, 10% iron trichloride for detection of tannins, Liebermann–Burchard reagent for triterpenoids, Draggendorff reagent for alkaloids, and iodine solution for starch [40]. All histochemical tests were compared with the respective unstained controls. The results were observed by LM, using an Olympus CX31 microscope coupled with a Leica MC170 HD digital camera controlled by the Leica Application Suite Version 4.8.0 software (Leica Microsystems Ltd., Heerbrugg, Switzerland).

### 4.7. Quantitative Analysis

The area of the cells of phellem, parenchyma, sclereid, and medullary rays and the area of secretory ducts, calcium oxalate druses, and starch grains were determined using the Leica Application Suite Version 4.8.0 software (Leica Microsystems Ltd., Heerbrugg, Switzerland). The number of medullary rays’ cells was also quantified.

### 4.8. Chemical Studies

#### 4.8.1. Extract Preparation

Aqueous extracts of red and white AoBs were prepared by maceration of the dried plant material in water (1:7 *w*/*v*) for 48 h, between 2 and 8 °C. The extracts were filtered using cotton tissue (according to the traditional way of preparing the THP) and lyophilized.

#### 4.8.2. Thin-Layer Chromatography

The chemical profile of AoB was established by TLC, using aqueous extracts of red and white AoBs, TLC cellulose plates as the stationary phase, and n-butanol-acetic acid-water (4:1:5, *v*/*v*/*v*) as the mobile phase [41,42], based on previous experimental data [25]. A total of 10 mL of each sample was spotted. After migration, each plate was sprayed with NEU reagent (previously prepared with 10 mL of 1% methanolic diphenylboric acid-β-ethylamino ester and 8 mL of 5% ethanolic polyethylene glycol-4000), and the chromatogram obtained visualized with UV light (366 nm). Gallic acid was used as a standard, and TLC co-chromatography also confirmed its presence in the sample. Ten different samples of red and white AoBs were analyzed, and three replicates of each were performed. The sequence of the zones present in the chromatograms obtained with the samples and the standard was registered; the R*_f_* of each zone was calculated.

#### 4.8.3. LC-UV/DAD Analysis

The chemical composition analysis of the red and white AoBs was performed by LC-UV/DAD, as described by Encarnação et al. (2022) [14].

#### 4.8.4. Quantification of Secondary Metabolites by Spectrophotometry

To ensure the validity of the results, all values were obtained in 3 sets of experiments and evaluated in triplicate using a Hitachi U-2000 UV-Vis spectrophotometer (Tokyo, Japan). Results were presented as a mean ± standard error of the mean (SEM).

- Total phenolic content

The total phenolic content was determined using a modified Folin–Ciocalteu method [43], previously validated by our team [17]. Increasing gallic acid concentrations (25–100 µg/mL) were used to obtain a standard curve, and the results were expressed as milligrams of gallic acid equivalents (GAE) per gram of dried AoB (mg GAE/g dried AoB). The total phenolic content was calculated using the following equation based on the standard curve: y = 0.0086x + 0.1045 and R^2^ = 0.99, where y is the absorbance, and x is the µg of GAE per µg of AoBTHP.

- Hydrolyzable Tannin Content

The hydrolyzable tannin content was determined according to the potassium iodate assay described by Willis and Allen (1998) [44], with some modifications. An aliquot of 1.5 mL of a saturated potassium iodate solution was added to the mixture of 1 mL of extract/water/standard solution with 2.5 mL of 80% acetone. After 40 min of incubation at 0 °C, the absorbance of the red intermediate was spectrophotometrically measured at 550 nm against the blank. Increasing gallic acid concentrations (200–800 µg/mL) were used to obtain a standard curve, and the results were expressed as milligrams of GAE per gram of dried AoB (mg GAE/g dried AoB). The hydrolyzable tannin content was calculated using the following equation based on the standard curve: y = 0.0007x + 0.0902 and R^2^ = 0.99, where y is the absorbance, and x is the µg of GAE per µg of AoBTHP.

- Condensed Tannin Content

The condensed tannins content was determined according to the butanol/hydrochloric acid assay described by Porter et al. (1986) [45], with some modifications. An aliquot of 5 μL of an acidic solution of ferrous sulfate (77 mg of iron (II) sulfate heptahydrate dissolved in 500 mL of 2:3 hydrochloric acid/n-butanol) was added to 0.5 mL of extract/water/standard solution. The tubes were loosely covered and placed in a water bath at 95 °C for 15 min. The absorbance of samples was spectrophotometrically measured at 530 nm against the blank. Increasing catechin concentrations (800–4000 µg/mL) were used to obtain a standard curve, and the results were expressed as milligrams of catechin equivalents (CAE) per gram of dried AoB (mg CAE/g dried AoB). The condensed tannin content was calculated using the following equation based on the standard curve: y = 0.0001x + 0.067 and R^2^ = 0.99, where y is the absorbance, and x is the µg of CAE per µg of AoBTHP.

- Total Triterpenoid Content

The total triterpenoid content was determined according to the colorimetric procedure described by Chang et al. (2012) [46]. An aliquot of 100 μL of extract/water/standard solution was mixed with 150 μL of vanillin/glacial acetic acid (5% *w*/*v*) and 500 μL perchloric acid solution. The sample solutions were heated for 45 min at 60 °C and then cooled in an ice water bath to the ambient temperature. After adding 2.25 mL of glacial acetic acid, the absorbances of the samples were spectrophotometrically measured at 548 nm against the blank. Increasing oleanolic acid concentrations (200–1000 μg/mL) were used to obtain a standard curve, and the results were expressed as milligrams of oleanolic acid equivalents (OAE) per gram of dried AoB (mg OAE/g dried AoB). The total triterpenoid content was calculated using the following equation based on the standard curve: y = 0.0003x + 0.0655 and R^2^ = 0.99, where y is the absorbance, and x is the µg of OAE per µg of AoBTHP.

### 4.9. Tests

#### 4.9.1. Loss on Drying

The loss on drying test of red and white AoBs was performed according to the standard method described in the European Pharmacopoeia 11.0 [38].

#### 4.9.2. Total Ash

The total ash test of red and white AoBs was performed according to the standard method described in the European Pharmacopoeia 11.0 [47].

### 4.10. Statistical Analysis

Data were analyzed using Microsoft Excel Version 16.49 software (Microsoft Corporation, Redmond, WA, USA) and GraphPad Prism Version 5.0 for Windows (GraphPad Software Inc., San Diego, CA, USA). Values are presented as mean ± SEM and were evaluated using the *t*-test. Differences were considered statistically significant when the *p*-value was lower than 0.05.

## 5. Conclusions

This work shows, for the first time, the botanical identification of the red and white AoBs as raw plant materials for pharmaceutical use, according to their macro- and microscopic characteristics.

We conclude that the areas of sclereid cells with thin cell walls, calcium oxalate druses, and starch grains can be helpful in the differentiation between red and white AoB types. The TLC profiles of red and white AoBs can also be considered for discriminatory purposes, and gallic acid can be used as a marker compound for quality control.

Although molecular biology could be an alternative way to perform AoB identification of both types, its assessment is expensive, and it has not yet been developed to identify these raw plant materials. Therefore, the botanical, chemical, and physicochemical results now presented and discussed are useful in this context and can be used as authentication and purity criteria for both types of AoBs.

## Figures and Tables

**Table 1:** Dimensions of *A. occidentale* bark examined by the naked eye.

Dimension (cm)	Red AoB			White AoB		
	Min ^a^–Max ^b^	Mean	SEM ^c^	Min–Max	Mean	SEM
Length	21.0–32.5	26.2	2.4	21.3–37.2	28.0	4.8
Width	2.2–4.8	3.3	0.6	2.4–7.0	4.5	1.3
Thickness	0.1–0.2	0.1	0.0	0.1–0.5	0.3	0.1

^a^ Min, minimum; ^b^ Max, maximum; ^c^ SEM, standard error mean.

**Table 2:** *A. occidentale* bark anatomical characteristics quantification.

Anatomical Characteristic	Red AoB (μm^2^)			White AoB (μm^2^)		
	Min ^a^–Max ^b^	Mean	SEM ^c^	Min–Max	Mean	SEM
Area of phellem cells	266.68–1771.56	994.93	51.65	71.81–11679.06	1224.61	464.35
Area of parenchyma cells	89.20–2172.55	915.46	66.23	8.16–12746.67	1208.61	195.39
Area of sclereid cells with thick cell walls	84.84–3672.37	894.44	101.54	20.24–2433.64	707.71	83.34
Area of sclereid cells with thin cell walls	62.73–3041.63	1370.51 *	100.89	5.75–4350.91	955.27	117.21
Area of secretory ducts	984.14–21934.02	7076.19	1145.72	90.96–29925.98	7957.99	1260.12
Area of medullary rays’ cells	2.19–2688.02	609.67	55.63	17.22–2820.15	664.51	65.41
Area of the calcium oxalate druses	13.31–629.41	279.32 *	34.04	55.78–7303.00	575.17	102.03
Area of the starch grains	0.65–44.35	9.43 *	1.54	1.88–191.92	18.25	2.73

^a^ Min, minimum; ^b^ Max, maximum; ^c^ SEM, standard error mean; * *p* < 0.001 vs. white AoB.

**Table 3:** Histochemical analyses of *A. occidentale* stem bark.

Compounds	Red AoB	White AoB	Coloration	Distribution
Alkaloids	-	-	-----	-----
Phenols	+	+	Reddish-purple	Major compounds on cortical parenchyma
Starch	+	+	Violet	Mainly in parenchymatous cells
Tannins	+	+	Blue and brownish colorations	Heterogeneous distribution
Triterpenoids	+	+	Reddish-purple	Mainly in thick-walled sclereids

+ Present; - Absent.

**Table 4:** Spectrophotometry quantification of secondary metabolites on *A. occidentale* stem barks.

Content Assay	AoB Extract	
	Red Type	White Type
Condensed Tannins (mg CAE/g AoB)	143.69 ± 4.67 *	73.79 ± 4.46
Hydrolyzable Tannins (mg GAE/g AoB)	9.38 ± 1.65 *	20.50 ± 1.00
Total Phenols (mg GAE/g AoB)	31.39 ± 0.50	31.36 ± 0.54
Total Triterpenoids (mg OAE/g AoB)	49.18 ± 0.82 *	62.18 ± 1.53

CAE: catechin equivalents; GAE: gallic acid equivalents; OAE: oleanolic acid equivalents; * *p* < 0.0001 vs. *A. occidentale* white.

## Data Availability

Not applicable.
